# Candidacidal Activity of a Novel Killer Toxin from *Wickerhamomyces anomalus* against Fluconazole-Susceptible and -Resistant Strains

**DOI:** 10.3390/toxins10020068

**Published:** 2018-02-03

**Authors:** Laura Giovati, Claudia Santinoli, Elena Ferrari, Tecla Ciociola, Elena Martin, Claudio Bandi, Irene Ricci, Sara Epis, Stefania Conti

**Affiliations:** 1 Department of Medicine and Surgery, University of Parma, 43125 Parma, Italy; laura.giovati@unipr.it (L.G.); claudia.santinoli@libero.it (C.S.); elena.ferrari@unipr.it (E.F.); tecla.ciociola@unipr.it (T.C.); 2 Department of Biosciences, University of Milan, 20133 Milan, Italy; elena.martin@unimi.it (E.M.); claudio.bandi@unimi.it (C.B.); sara.epis@unimi.it (S.E.); 3 Pediatric Clinical Research Center Romeo and Enrica Invernizzi, Ospedale “Luigi Sacco”, 20157 Milan, Italy; 4 School of Biosciences and Veterinary Medicine, University of Camerino, 62032 Camerino, Italy; irene.ricci@unicam.it

**Keywords:** *Wickerhamomyces anomalus*, yeast killer toxin, *Candida albicans*, *Candida glabrata*, antifungal resistance, A novel killer toxin produced by the recently isolated *Wickerhamomyces anomalus* strain 1F1 showed a differential killing ability against fluconazole-susceptible and -resistant *Candida* clinical isolates and laboratory strains displaying known mutations.

## Abstract

The isolation and characterization from the sand fly *Phlebotomus perniciosus* of a *Wickerhamomyces anomalus* yeast strain (*Wa*1F1) displaying the killer phenotype was recently reported. In the present work, the killer toxin (KT) produced by *Wa*1F1 was purified and characterized, and its antimicrobial activity in vitro was investigated against fluconazole- susceptible and -resistant clinical isolates and laboratory strains of *Candida albicans* and *C. glabrata* displaying known mutations. *Wa*1F1-KT showed a differential killing ability against different mutant strains of the same species. The results may be useful for the design of therapeutic molecules based on *Wa*1F1-KT and the study of yeast resistance mechanisms.

## 1. Introduction

Yeast strains belonging to diverse species produce and secrete proteins or glycoproteins, known as killer toxins (KTs), that are lethal to susceptible strains [1,2]. This property, which offers a competitive advantage to self-immune killer yeasts in their ecological niches, has found several applications in the biological control of plant pathogens and spoiling yeasts in the food and fermentation industries [3]. In the medical field, KTs have been used for the biotyping of pathogenic microorganisms, in epidemiological studies, and for the identification of novel cellular targets in microbial cells and the development of new antimicrobials [4,5,6].

Some KTs, such as K1 and K28 from *Saccharomyces cerevisiae*, have a narrow spectrum of activity, limited to susceptible strains of the same species, while other KTs show a wide killing spectrum [2]. In particular, some *Wickerhamomyces anomalus* (formerly *Pichia anomala*) KTs proved to be active against a wide range of microorganisms, including other yeast species, filamentous fungi, bacteria, and protozoan parasites [2,7,8].

Killer strains of *W. anomalus* have been isolated from different sources, including plants and food products [9,10,11], arthropods such as the crab *Portunus trituberculatus* [12], and mosquitoes of the species *Anopheles stephensi* [13,14]. Recently, the isolation and characterization of a *W. anomalus* strain displaying the killer phenotype was reported from specimens of the sand fly *Phlebotomus perniciosus* [15].

The ability to inhibit harmful microorganisms in a variety of habitats and the wide killing spectrum of the produced KTs have prompted the use of *W. anomalus* as a bio-control agent [7,16], since it could be classed as a low risk microorganism, rarely traced in human samples [17,18]. In addition, *W. anomalus* KTs activity against dermatophytes and pathogenic yeasts, especially *Candida* spp., led to the hypothesis that could be applied in medical mycology as alternative antifungal compounds [19,20,21].

*Candida* spp. are the most frequently isolated yeasts in clinical specimens. The frequency of invasive opportunistic fungal infections caused by species of this genus has significantly increased in recent years, particularly in immunosuppressed individuals and patients with indwelling medical devices [22]. Worldwide, the prevalent cause of invasive candidiasis remains *C. albicans*, although the epidemiology of candidal infections has gradually shifted towards non-*albicans* species, such as *C. glabrata* and *C. krusei* [22,23,24]. Increasing concern is rising in view of growing reports of resistance to antifungal drugs, with particular reference to resistance to azoles in non-*albicans Candida* species [25,26].

In the aim of searching for new molecules, potentially effective against strains resistant to conventional antifungal drugs, we investigated the in vitro activity of a KT produced by the recently isolated *W. anomalus* strain 1F1 (*Wa*1F1-KT) [15] against both susceptible and azole-resistant clinical isolates as well as laboratory strains of *C. albicans* and *C. glabrata* displaying known mutations [27,28,29,30,31].

## 2. Results

### 2.1. Wa1F1-KT Production

**Figure 1:**
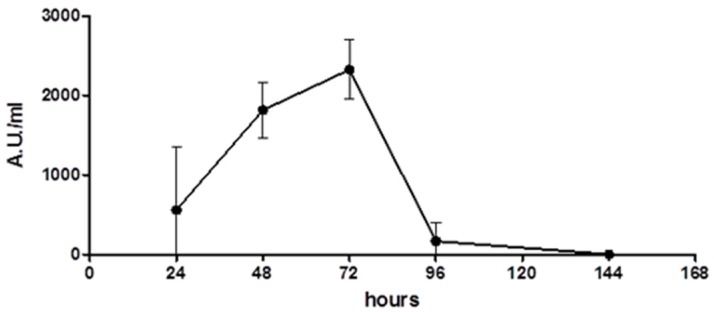
Time-course of *Wa*1F1-KT production by *Wickerhamomyces anomalus* 1F1. Overnight grown liquid seed cultures of the yeast were inoculated at 1% (*v*/*v*) into killer toxin (KT) production medium, then incubated at 20 °C under shaking (180 rpm). Samples were withdrawn at 24 h intervals, yeast cells were removed by centrifugation, and the filtered supernatants were concentrated 50-fold and assayed for their activity against the reference *Candida lusitaniae* strain grown on solid medium. Arbitrary Unit (AU): amount of KT which produces an inhibition zone of 1 mm^2^.

The production of *Wa*1F1-KT by the strain *W. anomalus* 1F1 was analyzed over time. The activity of the concentrated culture supernatant obtained at different time periods was determined against the reference *C. lusitaniae* strain on solid medium and quantified by Arbitrary Units/mL (1 Arbitrary Unit (AU) is defined as the amount of KT producing an inhibition zone of 1 mm^2^). As shown in Figure 1, the results indicate that the activity of the *Wa*1F1-KT sample obtained after 24 h of incubation was low (559 AU/mL), although it increased after 48 h of incubation (1815 AU/mL), and reached a maximum after 72 h (2326 AU/mL). The killing activity decreased steeply thereafter.

### 2.2. Western Blot Analysis of Wa1F1-KT

**Figure 2:**
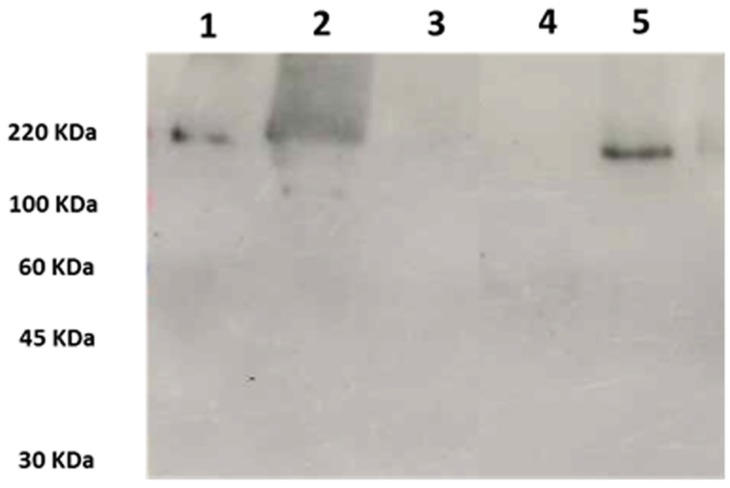
Western blot analysis using mAbKT4 to probe 50-fold concentrated crude extracts of (1) *Wickerhamomyces anomalus* ATCC 96603, 2 µL; (2) *W. anomalus* ATCC 96603, 5 µL; (3) *W. anomalus* UM3, 5 µL; (4) YPD medium, 5 µL; (5) *W. anomalus* 1F1, 5 µL. Molecular masses (kDa) are shown on the left.

Western blot analysis was carried out on crude extracts from 72-h cultures of the *W. anomalus* strains 1F1 and ATCC 96603 (KT-producing, positive control strain). As a negative control, a crude extract from *W. anomalus* UM3 (KT-nonproducing strain) culture and 50-fold concentrated YPD medium were used. Bands were detected with mAbKT4, a monoclonal antibody directed against a KT produced by the reference ATCC 96603 strain (*Wa*96603-KT) and shown to cross-react with KTs produced by other *W. anomalus* strains and other killer yeasts [14,32,33]. The results showed that mAbKT4 reacts with high-molecular mass proteins in samples obtained from *W. anomalus* 1F1 and ATCC 96603, but not from controls (Figure 2). A single band of approximately 220 kDa was revealed in extracts from the reference strain, while a single band with a lower molecular mass (approximately 160–170 kDa) was detected in the extract from 1F1 strain, indicating the secretion of KTs with a common epitope, although with some structural differences.

### 2.3. Characterization of Wa1F1-KT Using Size Exclusion Chromatography

**Figure 3:**
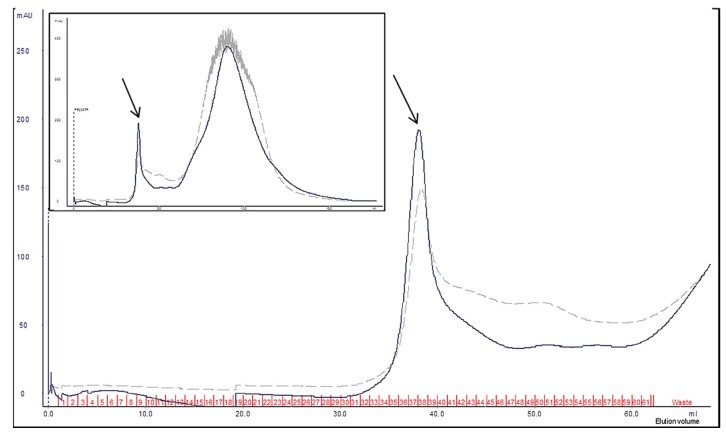
*Wickerhamomyces anomalus* 1F1 crude extract separation on HiPrep Sephacryl S-200 column. Partial chromatographic traces, displaying the active fractions (34–39), as determined by killing assay on *Candida lusitaniae* NEQAS 6208. Continuous and dashed lines refer to absorbance values (milli Absorbance Units, mAU) at 280 nm (mainly Trp absorbance) and 215 nm (peptide bond absorbance), respectively. Inset: complete chromatogram traces. The arrows indicate the elution peak of active fractions.

The concentrated extract from *W. anomalus* 1F1 culture was analyzed by size exclusion chromatography and eluted fractions were assayed for killing activity against the reference *C. lusitaniae* strain. In agreement with the immunoblot data, the results indicated the elution of active *Wa*1F1-KT in fractions 34–39 of the size exclusion chromatogram (Figure 3), corresponding to the highest molecular mass separation zone.

### 2.4. Activity of Wa1F1-KT

*Wa*1F1-KT was tested by an agar diffusion assay against the reference *C. lusitaniae* NEQAS 6208 strain and clinical isolates and laboratory strains of *C. albicans* and *C. glabrata* susceptible or resistant to fluconazole. The results showed that, when tested at 25 °C, *Wa*1F1-KT was active towards both fluconazole-susceptible and -resistant strains of *C. glabrata*, with some quantitative differences (Table 1). *Wa*1F1-KT showed no effect against the reference *C. albicans* SC5314 strain and against two fluconazole-susceptible clinical isolates (DSY347 and DSY544) and the DSY544-derived fluconazole-resistant mutant strain (DSY775) of *C. albicans*. On the contrary, a good effect was detected against the DSY347-derived fluconazole-resistant mutant *C. albicans* DSY289 strain. No effect against any of the tested strains was detected when the assay was carried out at 30 and 37 °C.

### 2.5. Exo-β-1,3-Glucanase Activity

**Figure 4:**
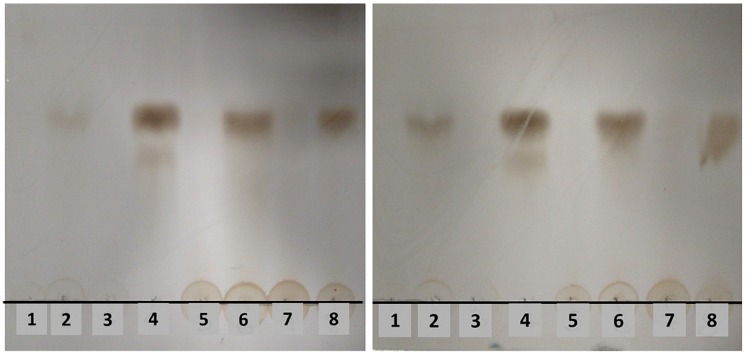
Thin-layer chromatogram of hydrolysis products of laminarin by crude killer toxins (KTs). Before loading, laminarin was incubated for 2 (left panel) or 4 (right panel) hours at 25 °C with: (1) heat-inactivated *Wa*1F1-KT; (2) *Wa*1F1-KT; (3) heat-inactivated laminarinase; (4) laminarinase; (5) heat-inactivated *Wa*96603-KT; (6) *Wa*96603-KT; (7) heat-inactivated *Wm*41968-KT; (**8**) *Wm*41968-KT.

To investigate if *Wa*1F1-KT could act on susceptible yeast strains by hydrolyzing major cell wall components, its ability to digest the soluble β-1,3-glucan laminarin was assayed in comparison with *Wa*96603-KT, the KT produced by the *Williopsis saturnus* var. *mrakii* MUCL 41968 (*Wm*41968-KT), with known β-glucanase activity [34], and laminarinase. The results showed that all KTs could hydrolyze laminarin to an end product presenting similar relative mobility to the product of the laminarinase reaction (Figure 4).

## 3. Discussion

Mucosal and invasive candidiasis are the most common mycoses in humans [22,23,24,35,36]. Invasive candidiasis and candidemia, in particular, are an emerging health problem, especially in hospitalized and immunosuppressed individuals and patients with indwelling medical devices [22]. Additionally, the diffusion of antifungal drugs resistance in *Candida* spp., particularly non-*albicans*, makes it necessary to look for new treatments against these infections [37].

In the present study, with the aim of searching for alternative antifungal agents, a KT produced by the newly isolated *W. anomalus* 1F1 strain [15] was assayed in vitro against clinical isolates and laboratory strains of *C. albicans* and *C. glabrata* displaying known mutations and different susceptibility to fluconazole [27,28,29,30,31].

KTs produced by strains of *W. anomalus* have previously shown to be active against *Candida* spp. both in vitro and in vivo [20,21,38,39], although little is known on their activity against resistant strains.

*W. anomalus* may produce different KTs with variable molecular mass (8–300 kDa), structural characteristics, pH and temperature optima, and antimicrobial activity range [7,40]. KTs from *W. anomalus* and other killer yeasts have been shown to exert a β-1,3-glucanase activity, and may cause damage to the cell wall of susceptible yeasts as a result of degradation of the main β-glucans cell wall components [20,34,41,42].

Our results showed that *Wa*1F1-KT shares at least one epitope with the KT produced by *W. anomalus* ATCC 96603, although the molecular mass of the two toxins is slightly different, indicating that they are related but not identical. Both *Wa*96603-KT and *Wa*1F1-KT degraded the soluble β-glucan laminarin in a manner similar to laminarinase, an endo-1,3(4)-β-glucanase, as did *Wm*41968-KT, whose glucanase activity had been already suggested [34].

The spectrum of activity of *Wa*96603-KT and *Wa*1F1-KT, however, appeared to be different, as the latter proved to be active against the *C. glabrata* isolates but did not affect the majority of *C. albicans* strains tested in this study, while *Wa*96603-KT was previously shown to kill different clinical *C. albicans* isolates [43,44]. Selective killing of non-*albicans* species by *W. anomalus* KTs with β-glucanase activity has previously been reported [20]. This differential spectrum may be explained with differences in the specificity of β-glucanase activity of KTs, which may selectively recognize different glycosidic linkages and glucan receptors on target yeast cells [45,46].

Although some KTs from *W. anomalus* display high stability at 37 °C and even higher temperatures [47], many KTs show lower thermostability [7]. We found that the optimal temperature for *Wa*1F1-KT activity was lower than the physiological value in the human body. Nevertheless, the absence of β-glucans on mammalian cells suggests its potential application against fungal infections at skin and mucosal membrane levels, as has been demonstrated with other KTs [48]. Further characterization of *Wa*1F1-KT enzymatic activity and the cloning of its encoding gene may represent the next step to investigate the feasibility to produce molecules with broader therapeutic activity, possibly including systemic infections.

The mechanism of action of *Wa*1F1-KT on *C. glabrata* appeared to be independent from the fluconazole-resistance pathway, as only slightly different effects were observed against the susceptible clinical isolate (DSY562) or its mutant derivative strains (SFY93, SFY105, SFY115, SFY116). This phenomenon underlies the potential of *Wa*1F1-KT as a universally active anti-*C. glabrata* tool, likely not affected by drug-resistance phenotypes.

On the contrary, the fact that *Wa*1F1-KT was active only on *C. albicans* DSY289 implies that the mechanism of action of the toxin may be dependent upon the specific mutations that confer resistance to fluconazole in this strain. *C. albicans* DSY289 was derived from the fluconazole-susceptible clinical strain DSY347 by mutations that confer combined resistance to azoles [27,29]. In particular, the point mutations S405F/Y132H in the *ERG11* gene encoding the enzyme lanosterol 14-α-sterol demethylase, which is involved in converting lanosterol into ergosterol, an essential component of the fungal cell membrane, are associated with a conformational change of the target enzyme and reduced interaction or binding of azoles [49,50,51]. The gain-of-function A736V mutation in the transcriptional activator *TAC1* causes the overexpression of the ATP binding cassette (ABC)-transporters *CDR1* and *CDR2*, decreasing the concentration of azoles within the fungal cell [29,52]. It is not clear how this mutation may affect *Wa*1F1-KT activity, but it may be speculated that the overexpression of transport systems and associated extracellular loops could possibly alter the recognition of the cell surface target by the toxin or that the transcriptional activator *TAC1* is involved in the regulation of transcription of toxin receptors.

Further studies aimed at the biochemical characterization of properly purified *Wa*1F1-KT, the elucidation of its mechanism of action, and the reasons for its differential killing ability against different mutant strains can provide important information for developing new strategies to combat infections caused by azole-resistant *Candida* strains.

## 4. Materials and Methods

### 4.1. Yeast Strains

Strains belonging to the species *W. anomalus*, *W. saturnus* var. *mrakii*, *C. lusitaniae*, *C. albicans*, and *C. glabrata* were used in this study. *W. anomalus* 1F1 isolated from *P. perniciosus* [15], *W. anomalus* ATCC 96603 (a KT-producing strain formerly referred to as UP25F) [32], and *Williopsis saturnus* var. *mrakii* MUCL 41968 [34] were used for the production of *Wa*1F1-KT, *Wa*96603-KT, and *Wm*41986-KT, respectively. The KT non-producing, KT-susceptible, *W. anomalus* UM3 strain was also used in this study as a negative control for KT expression [32].

The activity of *Wa*1F1-KT was tested against the reference *C*. *albicans* strain SC5314, two wild-type *C*. *albicans* clinical isolates (DSY544 and DSY347) [27,30], two *C*. *albicans* mutant strains resistant to fluconazole (DSY775, derived from DSY544, and DSY289, derived from DSY347) [27,29,30], two wild-type *C*. *glabrata* clinical isolates susceptible (DSY562) and resistant (DSY565) to fluconazole [28], and four *C. glabrata* fluconazole-resistant strains derived from DSY562 by mutations in the gene *CgPDR1* (SFY93, SFY105, SFY115, SFY116) [31]. The reference strain *C. lusitaniae* NEQAS 6208, known to be susceptible to the activity of *Wa*1F1-KT [15], was also used as a positive control for KT activity.

Yeasts maintained in sterile distilled water were subcultured on Sabouraud Dextrose Agar plates.

### 4.2. Media

KT-producing strains were grown in YPD medium (1% yeast extract, 2% peptone, and 2% dextrose), then subcultured for KT production in YPD medium with 15% glycerol, buffered at pH 4.6 with 0.1 M citric acid and 0.2 M Na_2_HPO_4_. For the KT activity assay, YPD medium was added with 3% agar, and 0.003% methylene blue, and adjusted to pH 4.6 with 0.1 M citric acid and 0.2 M Na_2_HPO_4_.

### 4.3. Production of KTs

For the production of crude extracts, a seed culture of the *W. anomalus* and *W. saturnus* var. *mrakii* strains was incubated at 20 °C for 24 h with shaking at 150 rpm in YPD medium. Flasks (500-mL volume) containing 100 mL of YPD buffered at pH 4.6, with 15% glycerol, were inoculated with 1 mL of the seed culture and incubated at 20 °C for 72 h with shaking (150 rpm). After this period, the cells were removed by centrifugation (5000× *g*, 10 min, 4 °C); the supernatant was filtered through 0.45 µm pore size membranes (Merck Millipore, Darmstadt, Germany) and concentrated (50-fold) through an Amicon Ultra-15 (10-kDa cutoff) filter unit (Merck Millipore) by centrifugation at 4000× *g*, 4 °C. Accordingly, a concentrated extract of YPD medium used for KT production was prepared. The concentrated crude extracts were stored at 4 °C until use.

### 4.4. Western Blot Analysis

The crude extracts from *W. anomalus* ATCC 96603, UM3, and 1F1 were analyzed by non-continuous denaturing sodium dodecyl sulfate polyacrylamide gel electrophoresis (SDS–PAGE) in 7% polyacrylamide gel, at 100 Volts for 2 h in a minigel system (Bio-Rad Laboratories, Hercules, CA, USA). Concentrated YPD medium was also run as a control.

Proteins were electrically transferred to a polyvinylidene difluoride (PVDF) membrane at 100 Volts for 1 h. The total protein content of the crude extracts was estimated by Ponceau staining prior to immunodetection. The PVDF membrane was then incubated for 1 h at room temperature with 5% bovine serum albumin in Tris-buffered saline (TBS) at pH 7.5 and 0.5% tween-20 (TBS-T). Subsequently, the membrane was incubated overnight at 4 °C with a 1:500 dilution in TBS-T of the monoclonal *W. anomalus* ATCC 96603 KT-neutralizing antibody mAbKT4 [32], known to cross-react with KTs from other *Wickerhamomyces* [14] and *Williopsis* [33] strains. After washing three times in TBS-T, the membrane was incubated for 1 h at room temperature with a secondary, peroxidase-conjugated anti-mouse antibody. The membrane was thoroughly washed with TBS-T, incubated for 1 min with the proper substrate (BM Chemiluminescence blotting substrate, Roche, Basel, Switzerland), and detected by ChemiDoc 2000R (Kodak, Rochester, NY, USA).

### 4.5. Characterization of Wa1F1-KT Using Size Exclusion Chromatography

The crude *Wa*1F1-KT was dialyzed against 0.01 M citric acid-Na_2_HPO_4_ buffer (pH 4.5) for 24 h at 4 °C using a membrane with a molecular mass cut-off of 10 kDa. Analytical gel filtration was performed on a HiPrep Sephacryl S-200 prepacked column (GE Healthcare Life Sciences, Marlborough, MA, USA), characterized by bed dimensions of 16 × 600 mm and an exclusion limit (for globular proteins) of about 400 kDa, connected to an AKTA purifier system (GE Healthcare Life Sciences). Dialyzed crude extract was applied to the column, equilibrated, and eluted with 1.2 column volume of citric acid-Na_2_HPO_4_ buffer, pH 4.5. Eluted fractions (1 mL) were combined according to chromatogram peaks, lyophilized, and re-solubilized in 1 mL of sterile distilled water. Total protein content was quantified with an infrared-based spectrometry system (Direct Detect™, Merck Millipore) and the concentrated fractions were assayed for killing activity against the *C. lusitaniae* NEQAS 6208 susceptible strain (see below).

### 4.6. Evaluation of Wa1F1-KT Activity

The activity of *Wa*1F1-KT was tested against the reference *C. lusitaniae* NEQAS 6208 strain and fluconazole-susceptible or -resistant clinical isolates as well as laboratory strains of *C. albicans* and *C. glabrata*. Each test strain, grown overnight on SDA plates, was resuspended in water to a final concentration of 0.5 McFarland and spread (100 µL) on the surface of YPD agar plates. Crude extracts from *W. anomalus* 1F1 were poured into wells of 8 mm (40 µL per well) cut into the agar plates. The plates were incubated for 48 h at 25, 30, or 37 °C and the diameter of the area of growth inhibition was measured. 

### 4.7. Laminarin Hydrolysis

The ability of *Wa*1F1-KT to hydrolyze the soluble β-1,3-glucan laminarin (Sigma-Aldrich, St. Louis, MO, USA) was assayed in comparison to *Wa*96603-KT, *Wm*41968-KT (with recognized β-glucanase activity [34]), and laminarinase (Sigma-Aldrich). The reaction mixtures contained 20 µL of 50-fold concentrated crude KTs or 10 µL of laminarinase (4.5 U/mL) and 2 mg/mL laminarin in 100 µL of citric acid-Na_2_HPO_4_ buffer (0.01 M, pH 4.5). After incubation at 25 °C for 2 and 4 h, the reaction was stopped by heating at 100 °C for 15 min. The activity on laminarin was estimated through observation of the end products of laminarin hydrolysis by thin layer chromatography [53]. Reaction mixtures containing crude KTs inactivated by heating at 100 °C for 15 min were used as controls.

## Figures and Tables

**Table 1:** Sensitivity of laboratory strains and clinical isolates of *Candida* spp. susceptible or resistant to fluconazole against killer toxin *Wa*1F1-KT.

Strain	Features and Genotype	*Wa*1F1-KT Sensitivity (mm Growth Inhibition) ^a^
*C. albicans* SC5314	Reference laboratory strain, FluS	0
*C. albicans* DSY347	FluS clinical strain [27]	0
*C. albicans* DSY289	FluR, DSY347 *ERG11*: S405F, Y132H; *TAC1*: A736V [27,29]	14
*C. albicans* DSY544	FluS clinical strain [30]	0
*C. albicans* DSY775	FluR, DSY544 *ERG11*: G464S, *TAC1*: G980W [30]	0
*C. glabrata* DSY562	FluS clinical strain [28]	13
*C. glabrata* DSY565	FluR clinical strain [28]	12
*C. glabrata* SFY93	FluR, DSY562 pdr1∆ [31]	12
*C. glabrata* SFY105	FluR, DSY562 pdr1∆-T588A [31]	13
*C. glabrata* SFY115	FluR, DSY562 pdr1∆-L280F [31]	13
*C. glabrata* SFY116	FluR, DSY562 pdr1∆-P822L [31]	13
*C. lusitaniae* NEQAS6208	Reference laboratory strain, FluS	17

^a^ Diameter (mm) of growth inhibition zone, mean values (±1 mm) from four independent experiments. FluS, Fluconazole-susceptible; FluR, Fluconazole-resistant.
